# Ophthalmia neonatorum complicated with neonatal orbital cellulitis: A case series

**DOI:** 10.51866/cr.438

**Published:** 2024-01-08

**Authors:** Ngee Ling Law, Vi Chee Tan, Thiam Hou Lim, Asyikin Nurul Rosli

**Affiliations:** 1 MD, MMed (Ophthal), Ophthalmology Department, Sibu Hospital, Jalan Ulu Oya, Sibu, Sarawak, Malaysia. Email: houlth@gmail.com; 2 MBBS, Ophthalmology Department, Sibu Hospital, Jalan Ulu Oya, Sibu, Sarawak, Malaysia.; 3 MBBS, Ophthalmology Department, Sibu Hospital, Jalan Ulu Oya, Sibu, Sarawak, Malaysia.; 4 MD, MRCPCH, Paediatric Department, Sibu Hospital, Jalan Ulu Oya, Sibu, Sarawak, Malaysia.

**Keywords:** Neonatal orbital cellulitis, Ophthalmia neonatorum, Delayed treatment

## Abstract

Orbital cellulitis is an extremely rare but potentially lethal condition in neonates. The clinical presentation of neonatal orbital cellulitis can be non-specific, and minimal signs of periorbital inflammation may go unrecognised by inexperienced parents or primary care medical personnel, leading to delayed treatment. Herein, we present a case series describing ophthalmia neonatorum complicated with neonatal orbital cellulitis owing to delayed treatment. The clinical presentation, management and outcomes are described. One neonate had orbital cellulitis, while the other had impending orbital cellulitis, with both cases resulting from delayed treatment of ophthalmia neonatorum. Both neonates were hospitalised for systemic antibiotic treatment and fully recovered with good outcomes. Timely identification and treatment of ophthalmia neonatorum are critical to mitigate potential severe sequelae, such as neonatal orbital cellulitis.

## Introduction

Neonatal orbital cellulitis is an extremely rare but potentially lethal condition.^1-3^ Its incidence varies widely across studies, which may be attributed to geographic and socioeconomic variations in diflerent populations. Sinusitis is reported as the most common predisposing factor of orbital cellulitis, accounting for up to 90% of cases.^4-6^ The infection may also spread from adjacent periorbital or facial infections, such as conjunctivitis, dacryocystitis, hordeolum, dental infection, direct inoculation from orbital trauma, animal or insect bite, ophthalmic surgery or periorbital skin infection.^6^ Premature birth and maternal infection during pregnancy may also increase the risk of developing neonatal orbital cellulitis.^2-4^

Ophthalmia neonatorum is a form of conjunctivitis occurring in the neonatal period.^7^ Its incidence ranges from 1% to 17% depending on the socioeconomic characteristics of the region. For example, the rates vary from 1% to 2% in the United States and Europe and to 17% in Pakistan.^7,8^ However, ophthalmia neonatorum complicated with orbital cellulitis is extremely rare. We hereby report two cases of orbital cellulitis in neonates, which resulted from delayed referral and treatment of ophthalmia neonatorum. We hope that this case series serves as a reminder for primary care physicians to treat ophthalmia neonatorum as an ocular emergency and promptly refer case to ophthalmologists upon the first encounter with the condition.

## Case presentation

### Case 1

**Figure 1:**
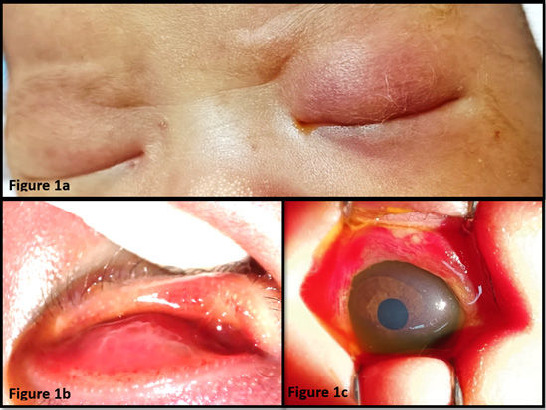
(a) Left erythematous swollen eyelid upon presentation. (b) Presence of pseudomembranes and associated meibomitis. (c) Left injected conjunctiva with 360-degree chemosis and subconjunctival haemorrhage, accompanied with minimal yellowish purulent discharge.

A full-term 14-day-old female neonate who was born via spontaneous delivery was referred from a polyclinic with a history of yellowish discharge from the left eye since day 9 of life. The condition was followed by left eyelid swelling and erythema on day 13 of life, which were accompanied with inconsolable crying for one night. The neonate remained active and tolerated feeding. Her mother was diagnosed with gestational diabetes mellitus under diet control and urinary tract infection (UTI) at 36 weeks of gestation. The neonate had a history of neonatal jaundice that did not require phototherapy. Her parents noticed her ocular problems and informed the medical officer during the first polyclinic follow-ups. However, the case was referred to an ophthalmologist only after the third visit to the polyclinic when the condition worsened. Upon receiving the referral, the ophthalmology team noticed that she was irritable during examination of her left eye. Ophthalmic examination of the left eye revealed an erythematous swollen eyelid with minimal yellowish discharge (Figure 1a). Pseudomembranes and associated meibomitis were noted (Figure 1b). The conjunctiva was severely injected and chemotic with subconjunctival haemorrhage. Furthermore, there was ophthalmoplegia at all gazes.

Nonetheless, the cornea was clear with a deep anterior chamber. The pupillary reflexes and fundus were normal. Right eye and systemic examinations showed unremarkable findings, and the neonate was afebrile. A sample of her eye discharge was sent for swab culture and sensitivity test, revealing no growth of organisms.

**Figure 2:**
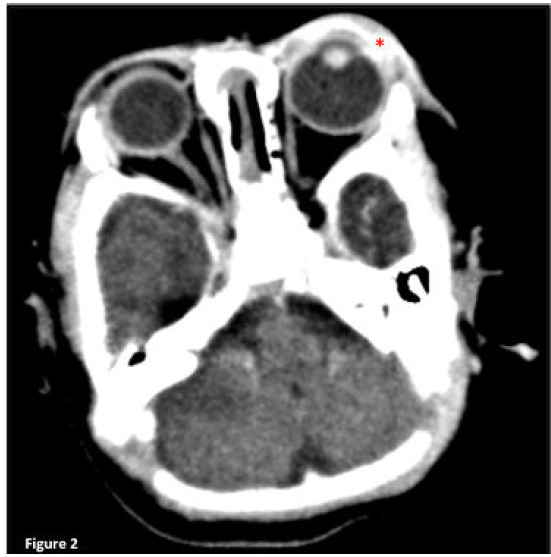
Computed tomography scan of the brain and orbit showing left periorbital subcutaneous fat stranding and thickening [*] with thickened left orbital septum and superior and inferior tarsal muscles.

Computed tomography (CT) of the brain and orbit was performed, which showed left orbital cellulitis with no evidence of orbital collection or intracranial extension (Figure 2).

**Figure 3:**
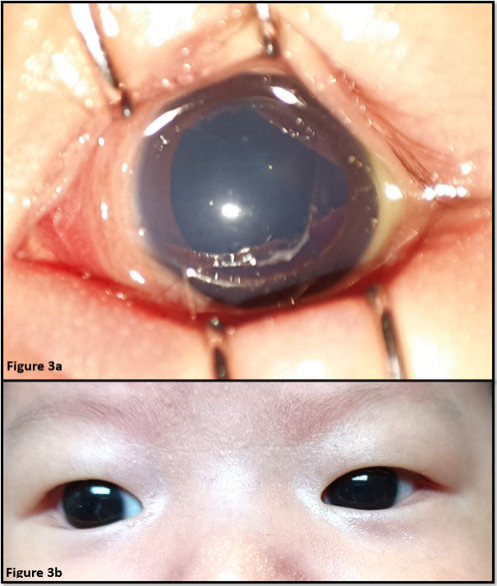
(a) Resolved left erythematous swollen eyelid with remaining minimal chemosis and slightly injected conjunctiva. (b) Fully recovered left orbital cellulitis with no chemosis or injection of the conjunctiva.

Intravenous cefotaxime 50 mg/kg/dose QID for 14 days and oral azithromycin 20 mg/kg/dose OD for 3 days were then administered. Guttae levofloxacin every 4 hour was added as ancillary therapy. Frequent eye toileting and daily removal of pseudomembranes were performed together with adjunct preservative-free artificial tear application, and low-dose guttae fluorometholone was added 3 days later. The neonate responded well to antibiotic therapy, as evidenced by reduced redness and swelling of the eyelid and the ability to open her eyes without aid. Upon completion of intravenous antibiotic therapy for 14 days, there was neither redness nor swelling of the eyelid with remaining minimal chemosis and slightly injected conjunctiva (Figure 3a). She was discharged home with guttae levofloxacin QID, and her orbital cellulitis fully resolved by the first week of follow-up (Figure 3b).

### Case 2

**Figure 4:**
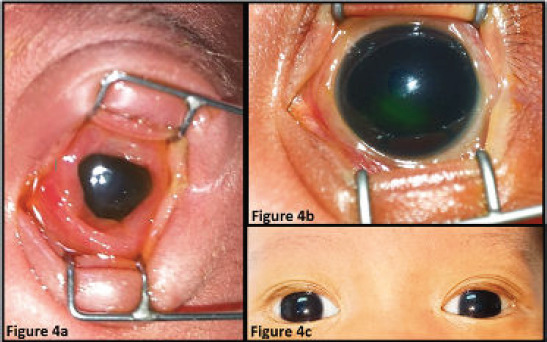
(a) Left severely injected conjunctiva with 360-degree chemosis and yellowish purulent discharge. (b) Resolving left conjunctival injection and chemosis after 7 days of treatment. (c) Fully recovered cellulitis with no chemosis or injection of the conjunctiva.

A full-term 10-day-old male neonate born via spontaneous delivery was referred to the ophthalmology clinic for left upper eyelid redness since day 7 of life, accompanied with yellowish discharge. The mother reported that she noted redness in his left eye since the first day of life but did not seek medical attention at that time. The neonate remained active and tolerated feeding. His mother had vaginal candidiasis at 38 weeks of gestation, and treatment was given. The neonate had neonatal jaundice that did not require phototherapy and was under polyclinic follow-up. Similar to case 1, the neonate’s ocular problems were not alerted by healthcare personnel at the early onset of the disease until third visit to the polyclinic because of a worsening condition. The neonate became irascible upon examination of his left eye. His left eyelid was erythematous and swollen, accompanied with yellowish discharge. His conjunctiva was severely injected and chemotic with the presence of pseudomembranes (Figure 4a). Moreover, his extraocular movements were restricted at all gazes.

**Figure 5:**
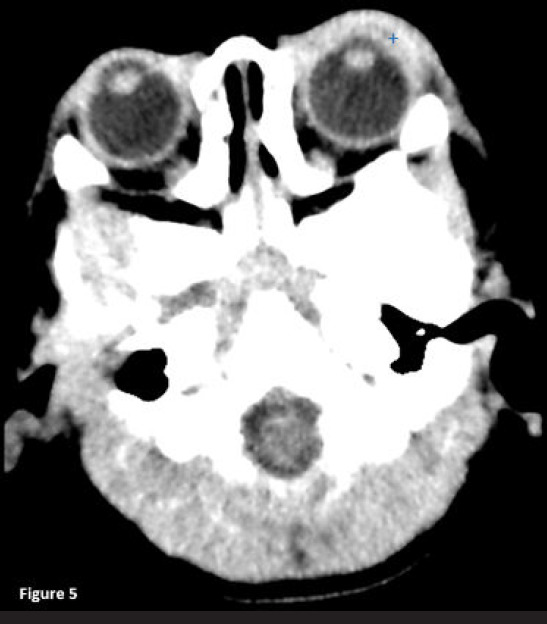
Computed tomography scan of the brain and orbit showing left periorbital soft tissue swelling with no fat stranding [+] representing left periorbital cellulitis.

Consequently, CT of the brain and orbit was performed, which revealed left-enhancing periorbital soft tissue swelling representing periorbital cellulitis with no evidence of orbital collection or intracranial extension (Figure 5). Left eye swab culture and sensitivity test demonstrated the growth of *Serratia marcescens.*

Given the clinical signs suggesting orbital cellulitis, intravenous cefotaxime, oral azithromycin and guttae levofloxacin were administered. The neonate responded well to treatment. His eyelid swelling and redness along with chemosis resolved more rapidly than did those of case 1 (Figure 4b). The neonate was discharged after completing 7 days of intravenous antibiotic treatment. He was prescribed with syrup cefuroxime 15 mg/ kg/dose BD and guttae levofloxacin for 1 week upon discharge. He fully recovered after the 2-week course of treatment (Figure 4c).

## Discussion

The prevalence of bacterial flora varies across different age groups owing to differences in their immunity and anatomical structures.^9^ In neonates, Staphylococcus and Streptococcus species are the most common causative agents of ophthalmia neonatorum.^6,8-10^ Conversely, Haemophilus species may be more common in older infants.^9^ Serratia species are occasionally recognised as a cause of hospital-acquired infection such as UTI, respiratory tract infection or wound infection.^11^ In this study, S. marcescens was cultured in case 2.

In the present cases, the infection may have started from vertical transmission from the mothers who had a history of UTI and vaginal candidiasis, leading to perinatal infection and, subsequently, ophthalmia neonatorum.^12^ Later, the delayed treatment of ophthalmia neonatorum may have contributed to the development of orbital cellulitis. A study conducted in Ilorin found that antenatal maternal vaginal discharge is also one of the predisposing factors of ophthalmia neonatorum.^12^

The clinical presentation of neonatal orbital cellulitis can be non-specific, which makes it challenging to diagnose. Simple fever, reduced milk intake, moaning or inconsolable crying, minimal eye discharge or mild conjunctival redness may present as the initial symptoms along with non-obvious periorbital swelling or erythema.^2^ If the minimal signs of periorbital inflammation are not recognised by inexperienced medical personnel in primary care settings, treatment may be delayed.^2^ Thus, it is crucial for primary care medical personnel to have a high level of suspicion and be aware of potential ocular problems, especially when informed by caretakers. Delayed treatment can lead to serious complications, as demonstrated in the present cases.

Ceftriaxone is commonly used as the first-line antibiotic for orbital cellulitis in patients of other ages. In the present cases, cefotaxime was administered, since ceftriaxone carries a higher risk of hyperbilirubinaemia in neonates if more than one dose is needed in the short term.^13,14^ Both ceftriaxone and cefotaxime are broad-spectrum antibiotics that cover a wide range of bacteria, including Streptococcus and Staphylococcus species, which are the most common causative organisms.^15^

Concurrently, we highly recommend regular eye toileting and frequent preservative-free artificial tear application as adjunctive therapy to locally remove the causative pathogen.^7^ Daily removal of pseudomembranes is proven to be helpful in accelerating the healing of the inflammatory state of conjunctivitis. Furthermore, low-dose topical steroids can add an adjuvant effect to shorten the recovery period by reducing inflammation.^16^

Early detection and prompt treatment are crucial for a favourable outcome of neonatal orbital cellulitis. In less severe cases, a shorter period of hospitalisation and faster recovery can be expected, as demonstrated in case 2. However, in severe cases, such as those with abscess formation or associated sepsis, a longer period of hospitalisation and more aggressive treatment such as surgical drainage may be required.^1,5,6,17,18^

Primary care teams play a crucial role in managing cases of ophthalmia neonatorum. Any instances of ophthalmia neonatorum, including eyelid swelling or redness, should be promptly referred to an ophthalmologist for thorough assessment and evaluation. It is important for primary care teams to be aware that ophthalmia neonatorum is an ocular emergency requiring immediate referral.^19^

Primary care team members, especially junior medical officers, are strongly encouraged to attend the ‘Primary Eye Care Course’ to enhance their foundational knowledge of basic eye care in primary care settings.

## Conclusion

Ophthalmia neonatorum is an ocular emergency requiring immediate referral by primary care teams to ophthalmologists upon the first encounter of eye discharge or redness in neonates for thorough assessment and management. 19 This proactive approach is crucial to prevent serious complications such as orbital cellulitis and reduce the risk of permanent eye damage or vision loss.^1,3,7,18-19^

## References

[1] Lavaju P, Badhu B, Khanal B, Shrestha B (2014). Orbital cellulitis in a neonate of the tooth bud origin: a case report.. Indian J Ophthalmol..

[2] Rong L, Liu Y, Cai C (2020). Clinical analysis of 6 cases of orbital cellulitis in neonates and literature review.. Chin J Appl Clin Pediatr..

[3] Lei TH, Huang YC, Chu YC, Lee CY, Lien R (2013). Orbital cellulitis caused by community-associated methicillin-resistant Staphylococcus aureus in a previously healthy neonate.. J Microbiol Immunol Infect..

[4] Santos JC, Pinto S, Ferreira S, Maia C, Alves S, da Silva V (2019). Pediatric preseptal and orbital cellulitis: a 10-year experience.. Int J Pediatr Otorhinolaryngol..

[5] Mallika PS, Tan Ak, Aziz S, Vanitha R, Tan TY, Faisal HA (2009). Orbital cellulitis complicated by subperiosteal abscess in a neonate with ethmoiditis.. HK J Paediatr (New Series).

[6] Durairaj V, Gonzalez M (2010). Understanding pediatric bacterial preseptal and orbital cellulitis.. Middle East Afr J Ophthalmol..

[7] Castro Ochoa KJ, Mendez MD (2023). Ophthalmia Neonatorum.. StatPearls..

[8] Puente MA (2021). Neonatal conjunctivitis (ophthalmia neonatorum).. Medscape..

[9] Molarte AB, Isenberg SJ, Palmer EA (1989). Periorbital cellulitis in infancy/discussion.. J Pediatr Ophthalmol Strabismus..

[10] Alsulaiman HM, Al-Faky Y (2021). Microbiology and outcome of pediatric orbital cellulitis in a tertiary eye care center in Saudi Arabia after the routine administration of Haemophilus influenzae type B vaccine.. Saudi J Ophthalmol..

[11] Khanna A, Khanna M, Aggarwal A (2013). Serratia marcescens- a rare opportunistic nosocomial pathogen and measures to limit its spread in hospitalized patients.. J Clin Diagn Res..

[12] Boadi-Kusi SB, Kyei S, Holdbrook S, Abu EK, Ntow J, Ateko AM (2021). A study of ophthalmia neonatorum in the central reion of Ghana: causative agents and antibiotic susceptibility patterns.. Glob Pediatr Health..

[13] Monte SV, Prescott WA, Johnson KK, Kuhman L, Paladino JA (2008). Safety of ceftriaxone sodium at extremes of age.. Expert Opin Drug Saf.

[14] Hile GB, Musick KL, Dugan AJ, Bailey AM, Howington GT (2021). Occurrence of hyperbilirubinemia in neonates given a short-term course of ceftriaxone versus cefotaxime for sepsis.. J Pediatr Pharmacol Ther..

[15] Shih EJ, Chen JK, Tsai PJ, Lin MC, Bee YS (2022). Antibiotic choices for pediatric periorbital cellulitis—a 20-year retrospective study from Taiwan.. Antibiotics..

[16] Ho D, Lim S, Kim Teck Y (2020). Pseudomembranous conjunctivitis: a possible conjunctival foreign body aetiology.. Cureus.

[17] Kaul A, Dhalait S, Shah S, Murthy R (2019). Orbital abscess in an infant: a rare presentation of sinusitis.. J Pediatr Crit Care..

[18] Bhandari AJ, Gogri PY, Misra SL, Misra NS, Gidwani HV (2015). Neonatal orbital abscess.. Oman J Ophthalmol..

[19] Mallika PS, Asok T, Aziz S, Faisal HA, Tan AK, Intan G (2008). Neonatal conjunctivitis - a review.. Malays Fam Physician..

